# Early Childhood Obesity Risk Factors: Socioeconomic Adversity, Family Dysfunction, Offspring Distress, and Junk Food Self-Medication

**DOI:** 10.1007/s13679-018-0310-2

**Published:** 2018-04-27

**Authors:** Erik Hemmingsson

**Affiliations:** 1 The Swedish School of Sport and Health Sciences, Box 5626, 114 86 Stockholm, Sweden; 2 Department of Medicine, Karolinska Institutet, Stockholm, Sweden

**Keywords:** Affective states, Emotion regulation, Family environment, Food addiction, Infancy, Obesity etiology

## Abstract

**Purpose of Review:**

To explore the sequence and interaction of infancy and early childhood risk factors, particularly relating to disturbances in the social environment, and how the consequences of such exposures can promote weight gain and obesity.

**Recent Findings:**

This review will argue that socioeconomic adversity is a key upstream catalyst that sets the stage for critical midstream risk factors such as family strain and dysfunction, offspring insecurity, stress, emotional turmoil, low self-esteem, and poor mental health. These midstream risk factors, particularly stress and emotional turmoil, create a more or less perfect foil for calorie-dense junk food self-medication and subtle addiction, to alleviate uncomfortable psychological and emotional states.

**Summary:**

Disturbances in the social environment during infancy and early childhood appear to play a critical role in weight gain and obesity, through such mechanisms as insecurity, stress, and emotional turmoil, eventually leading to junk food self-medication and subtle addiction.

## Introduction

Obesity is clearly one of the most pressing public health challenges of our time. A recent simulation study from the USA found that by 2030, a staggering 55–60% of today’s children will be obese [1]. Although incidence rates have generally slowed down or even reversed in populations with higher socioeconomic status (SES) [2, 3••, 4••], there are few clear signs that this general upward trend will be reversed anytime soon, especially for low SES groups [4••, 5]. The costs to society and individuals are already monumental, and are set to increase further as numbers affected and the duration of obesity exposure increase [6, 7]. Indeed, the current and predicted future situation is nothing short of a public health calamity, requiring urgent action from individuals, organizations, corporations, health care, and governments alike.

Obesity has proved resistant to conventional treatment, through adaptations such as reduced metabolic rate and an increase of appetite increasing hormones [8, 9]. Prevention is therefore an absolutely critical strategy, theoretically by reducing population exposure to common risk factors. Identification of these risk factors, and increased understanding about how those risk factors interact, is therefore vital.

Research shows that the ages between 0 and 5 years is a critical period in the development of overweight and obesity [10•, 11, 12], and that childhood overweight and obesity is highly predictive of adult obesity [1, 10•]. Early prevention efforts are therefore a clear priority [13], as is increased understanding about infancy and early childhood risk factors [8, 14].

The idea of childhood adversity as an independent obesity risk factor is gaining attention, where two relatively recent meta-analyses both found clear increases in adult obesity risk in children exposed to abuse or maltreatment [15, 16]. In addition, childhood adversity can also, through such consequences as low self-esteem, poor mental health, chronic stress, chronic inflammation, emotional turmoil, and increased appetite, influence the regulation of adipose tissue mass upwards (body weight set-point theory) for increased security and survival [8], and lead to redistributions of peripheral body fat to more visceral areas [17].

Earlier pioneering work in this field found a strong association between childhood neglect, a strong sign of a harsh social milieu (using data from teacher proxy report), and a subsequent obesity development [18]. Moreover, it is already well established that childhood abuse likewise drastically increases the risk of many of our leading causes of death, such as mental health problems, addiction, heart disease, stroke, several cancer forms, suicide, type 2 diabetes, and severe obesity [19]. In summary, the evidence is mounting for a potent role of a harsh social environment during infancy and early childhood in the etiology of obesity and several obesity comorbidities.

**Fig. 1:**
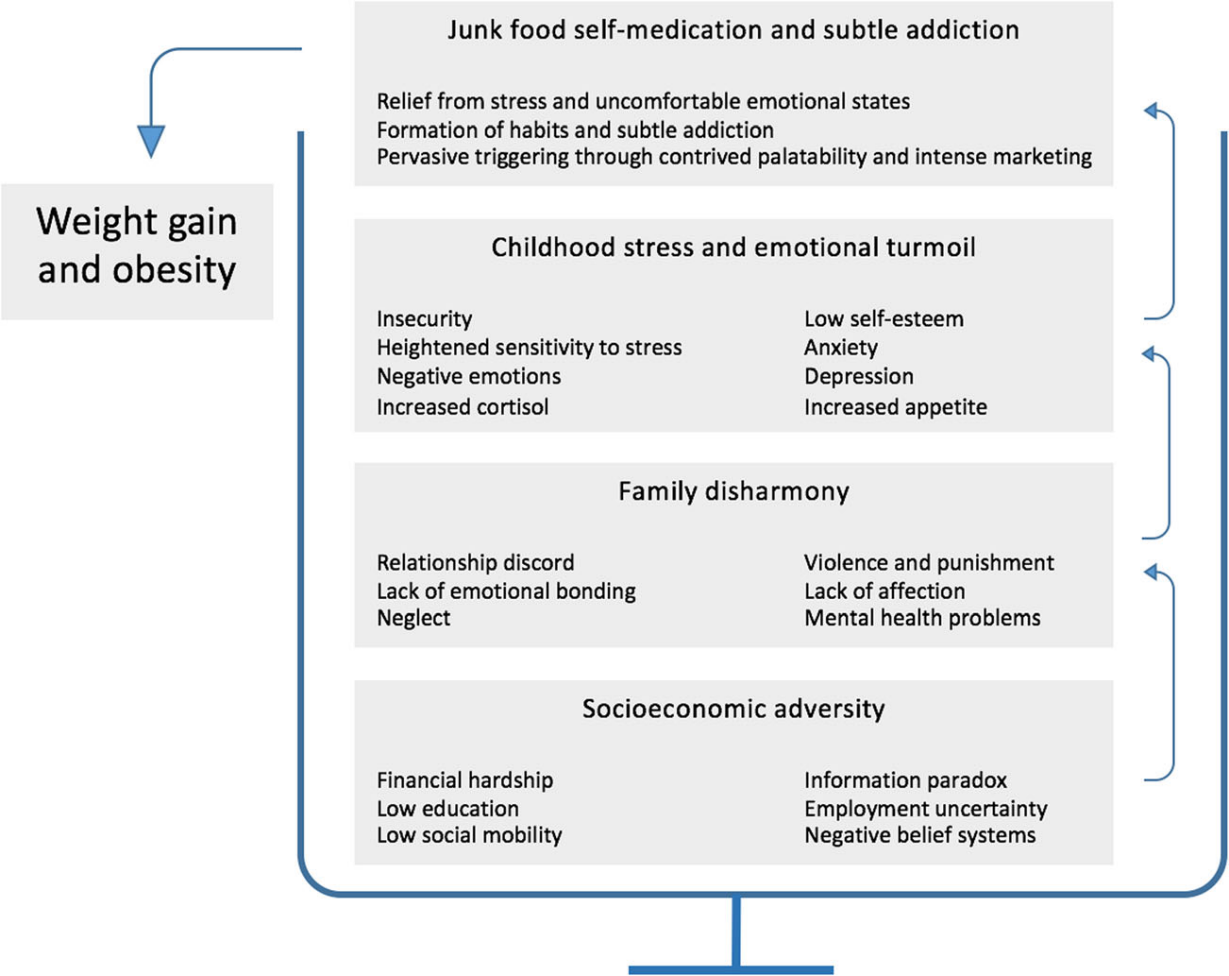
Sequencing and interaction of obesity risk factors particularly relating to social disturbances during infancy and childhood, increasing the risk for junk food self-medication, resulting in a disruption of energy homeostasis, i.e., weight gain and obesity (here illustrated as an overflowing cup)

While a previous review of this general topic explored the underlying reasons behind the low SES and obesity association [8], the current review will explore how upstream and midstream risk factors (childhood adversity, insecurity, stress, and emotional factors) interact with downstream risk factors, mainly calorie-dense junk food, gradually causing the cup to spill over (Fig. 1). Emphasis will also be placed on clarifying the strongly habit-forming and probably addictive properties of junk food, particularly for individuals with increased stress and emotional turmoil, as a result of a harsh family environment during early childhood.

## Socioeconomic Adversity

Having a low socioeconomic status is arguably one of the strongest risk factors for developing obesity (in countries that have made the transition to Western lifestyles) [2, 20, 21], and many other adverse health conditions, including diabetes, cancer, and mental health problems [22].

There is a growing body of literature suggesting that the association between socioeconomic status and development of childhood obesity is growing in strength with an ever widening gap in obesity rates between low and high SES groups [3••, 23–26]. In many developed countries, the rates of childhood obesity have stabilized or even been reduced in higher SES groups, whereas lower SES groups have generally seen a steady increase [3••, 23–26]. This clearly highlights the role of low SES as a critical risk factor. As the income gap continues to widen in our societies, we can expect the adverse influence of low SES to increase even further, particularly for countries where obesity is already well entrenched.

There are several main consequences of low SES that are of particular relevance to obesity: mental health (depression, anxiety), low self-esteem and self-worth, feeling disempowered, insecurity, stress, negative belief systems, and negative emotions (anger, apathy, hopelessness, frustration, shame, guilt, etc.) [27••, 28–31]. Another hallmark of low SES is financial hardship, making healthy lifestyle choices less accessible, indirectly promoting unhealthy lifestyle choices, such as consuming unhealthy and usually calorie-dense foods, a lack of physical activity, and less opportunity for healthy growth and development, such as education [25, 26]. Another stamp of low SES is a lack of higher education and critical thinking, which promotes susceptibility to junk food marketing [26, 32].

## Family Dysfunction

Starting a family and becoming a parent is a major life-changing event that drastically elevates demands and responsibilities, not least financially but also in the role as the primary provider of social, mental, and emotional development for children. Families where parents have low SES is not only characterized by financial lack, but often a lack of cohesion, low awareness or prioritization of healthy lifestyles, neglect, relationship discord, mental health problems, low self-esteem, and addiction, resulting in a harsh and insecure social environment for infants and young children [27••, 33].

In short, the desired social, psychological, and emotional nurturing of the child may be disturbed during some of its most critical developmental periods [27••]. It is well established that children growing up under these circumstances are at much increased risk of various adverse health outcomes relevant to obesity development, such as mental health problems, addiction, and chronic inflammation [34, 35]. Using a retrospective study design, Felitti et al. (Adverse Childhood Experiences Study) were one of the first to demonstrate the potent role of family dysfunction and adverse childhood experiences on numerous health outcomes [19]. The list of adverse childhood exposures included psychological, physical or sexual abuse, parental violence, substance abuse, mental illness, and imprisonment. More than half the respondents reported at least one adverse exposure, with a quarter reporting at least two adverse exposures. There were consistent and graded associations between adverse childhood experiences and outcomes such as alcoholism (OR: 7.4), (comparing four or more exposures vs none), drug injection (10.3), mental illness (4.6), attempted suicide (12.2), bronchitis (3.9), ischemic heart disease (2.2), stroke (2.4), smoking (2.2), poor self-rated health (2.2), and severe obesity (OR: 1.6), all statistically significant. The consistency and strength of these associations clearly highlight a very toxic role of childhood abuse in adult health development.

## Offspring Insecurity, Stress, and Emotional Turmoil

Infancy and early childhood is when humans are most vulnerable and reliant on external nurturing (including breastfeeding), security, attachment, and support, since young children have not yet acquired adequate protective mechanisms (resilience, coping skills, self-esteem, independence, etc.).

Since the path to obesity usually starts during early childhood [4••, 10•], combined with the inverse association between SES and obesity, we need to understand more about the effects of family adversity on the offspring. Infants and children are highly impressionable, and tend to internalize much of their external environment, thereby increasing the risk of uncomfortable psychological and emotional states as a result of family stress and strain [36].

Consequences of a dysfunctional infancy and childhood include an exaggerated sensitivity to social, physical, and emotional stressors (slamming doors, harsh language, violence, social distance, lack of warmth and affection, neglect, etc.), resulting in [37] insecurity and anxiety, mental health problems, negative emotions (fear, anger, apathy, abandonment, hopelessness, disappointment, boredom, grief, shame, emptiness, guilt, bitterness, jealousy, tiredness, self-pity, resigned, etc.), behavioral and learning problems, low self-esteem, low self-worth, low self-efficacy, sleeping problems, etc. [8, 27••, 38, 39•]. The emotional toll of exposure to verbal or physical aggression between parents has been shown in prospective studies in infants and young children [37, 40]. Without support from other important others or adequate resilience, such exposure will result in more or less chronic stress (elevated cortisol and ghrelin), chronic inflammation and insecurity and uncomfortable emotional states [8, 27, 33, 38, 39•, 41, 42].

## Formation of Junk Food Self-Medication Habits and Subtle Addiction

Once stress, insecurity, and emotional turmoil have been established at an early age, the individual will naturally seek relief from these uncomfortable states, with little interference from cognitive processes, through the brain-reward system [17, 43]. Junk food, given its hedonic properties through an exaggerated energy density [44, 45], is a readily available form of self-medication through hedonic binge eating, creating strong habits through changes in the amygdala and hippocampus [17, 46–49]. While the concept of food addiction is debated [50], the term has nevertheless gained some recognition, with one study reporting 23% of children 5–12 years (data collected through parent proxy), being classified as addicted to food, and a positive association with obesity [51].

Children in disharmonious families are therefore at greater risk, compared to children who grew up in harmonious families, to rapidly adopt effective but ultimately dysfunctional eating habits where they regularly consume energy dense junk food for emotional and stress-related relief and pleasure. [17] Once such habits have been established, it is unlikely to change as a result of cognitive interference [17, 47, 48].

## Untangling a Fundamentally Flawed Internal/External Environment Interaction

Highly palatable and emotionally rewarding junk food is pervasive in our external environment, constantly providing cues for consumption much beyond our metabolic need. Obese individuals have been shown to be more affected than normal weight individuals by external cues, feel stronger cravings, and consume greater portions after exposure [52]. Research also suggests that the hedonic responses and adaptations in the brain from eating palatable junk food are similar to more conventional drug abuse [43, 53].

Even though the triggered consumption of junk food through internal factors like stress, elevated ghrelin levels, and uncomfortable emotions, is not as potent as more chemically induced addiction, junk food can nevertheless be described as a type of subtle and arguably more insidious type of addiction or addictive-like behavior [52]. This is both good and bad for the individual: cravings and withdrawal symptoms are not as powerful, but this type of addictive behavior may also be harder to detect and counter, for example through therapy.

Another vastly complicating factor is that junk food, compared to regular drugs, is much more available, cheaper, and marketed extensively. In addition, since food is necessary to our survival, there exist infinitely more opportunities to trigger a level of hedonic food consumption much beyond our metabolic and nutritional need, for example through adding sugar, fat, and salt to increase palatability and initiate binge-like eating behavior.

In summary, many individuals of varying ages today find themselves with unresolved internal stress and uncomfortable emotions, particularly during demanding or adverse living conditions, existing in an external environment that is more or less constantly inviting a quick, cheap, and highly available solution: energy dense ultra-processed junk food, inviting a more or less addictive, emotionally triggered eating behavior.

## Improving Treatment and Prevention Efforts

An important historical lesson that needs to be considered is that risk factors like low SES and emotional turmoil have existed long before the rise of obesity. However, drawing on the filling-of-the-cup metaphor (Fig. 1), the obesity cup was not spilling over until the introduction and growth of ultra-processed junk food in all its many different forms from the 1960s and onwards [54]. It is therefore reasonable to propose that low SES and emotional turmoil merely set the stage for weight gain and obesity, whereas the introduction of energy-dense junk food can be considered a direct cause of weight gain and obesity through its influence on energy homeostasis.

Prevention of obesity has been tried, although given the current toxicity of the external environment, combined with unresolved stress and negative emotions in a large segment of the population, these efforts (while laudable) have been limited in scope. Hitherto, the main prevention strategies have been raising public awareness, improved diet and increased physical activity [55]. Given that there are many examples of obesity rates that have generally slowed or even reversed in high SES strata [2, 3], one could argue that such simple strategies have had some success in affluent and well educated populations, quite possibly due to less exposure to socioeconomic adversity, stress, and emotional turmoil.

What remains a much greater challenge is to prevent obesity for low SES groups, particularly children in dysfunctional families. This is arguably where the need for prevention is greatest, and where considerable more effort urgently needs to be mobilized, for example in schools and kindergartens and families under financial and social strain [56, 57]. In theory, we could improve outcomes by focusing more on upstream and midstream risk factors, such as reducing poverty, increasing education, and providing support for families, combined with more traditional downstream drivers, i.e., more balanced nutrition, reduced junk food marketing, and increased physical activity.

Given the general long-term failure of calorie-centered diet and exercise schemes, and the classic pattern of weight loss and regain [9], suggesting the presence of a body weight set-point [8] it is urgent that obesity treatment programs delve deeper into psychological and emotional aspects so that weight loss maintenance can be improved. Treatment outcomes for obesity could be improved by targeting the causes behind excessive eating, such as maladaptive junk food self-medication habits formed during childhood or other stressful and emotional events thereafter (losing a job, divorce, grief, etc.).

It is here speculated that these inner perturbations, mainly emotional, and related junk food habits and associated addiction/self-medication are much more common than many clinicians and patients realize, indicating a need for more research in this promising area to find more definitive solutions to obesity. Obtaining data on infancy and early childhood social stressors is challenging, although not impossible, for example, through retrospective investigations, [15, 16] teacher or relative proxy, [18] or register-based investigations [58–60].

## Conclusions

Infancy and early childhood is a critical period in the development of overweight and obesity. There appears to be a highly toxic trio of risk factors in socioeconomic adversity (upstream), offspring stress and emotional turmoil (midstream), and subtle junk food self-medication to alleviate uncomfortable emotional states (downstream). Over time, this results in energy homeostasis disruptions, weight gain, and obesity. The effectiveness of childhood prevention efforts may be improved by including more upstream and midstream risk factors, as a complement the more established improved diet and exercise programs.
